# P2Y_11_ receptor is a critical regulator of extracellular ATP-mediated premature senescence in lung fibroblasts: Implications of ER-Ca^+2^ release/mitochondrial ROS production signaling pathway

**DOI:** 10.1007/s11302-024-10036-9

**Published:** 2024-07-08

**Authors:** Abdel-Aziz S. Shatat

**Affiliations:** Department of Pharmacology and Toxicology, Faculty of Pharmacy, Al-Azhar University, 11751 El Nasr St, Nasr City, Cairo Egypt

**Keywords:** Premature senescence, Lung fibroblasts, Extracellular ATP, P2Y11 receptor

## Article summary

Cellular senescence is characterized by the cessation of cell division. Numerous stressful stimuli, such as UV light exposure and oxidative stress, can hasten senescence [1]. Nucleotides and their purinergic receptors (P2R) have been reported to play a central role in many different cellular signaling processes. In a recent publication in *J. Biol. Chem*, Volonte et al*.* [2], showed that oxidative stress mediated ATP release and induced human lung fibroblast senescence. Extracellular ATP or oxidative stress-mediated premature senescence was inhibited by pharmacological antagonism of the P2Y11 receptor (P2R_11_R) with a selective antagonist, NF-157; furthermore, the P2Y_11_R activation with NF-546 (a selective P2Y_11_R agonist) induced cellular senescence. In addition, P2Y_11_R activation by NF-546 or ATP evoked endoplasmic reticulum (ER) Ca^+2^ release, which subsequently entered mitochondria and induced reactive oxygen species (ROS) generation. This in turn prompted premature senescence of lung fibroblasts. Finally, the authors demonstrated that ATP-stimulated lung fibroblasts secreted amphiregulin, which enhanced the growth of triple-negative breast cancer cells.

## Commentary

The senescence-clearance-regeneration model of cells involves three processes: stable proliferative arrest, secretory phenotype, and mobilization of progenitors for tissue remodeling [3]. The senescence-associated secretory phenotype refers to the profusion of cytokines, growth factors, and proteases secreted by senescent cells [4]. Senescent cells play opposing, multifaceted roles in cancer, as cellular senescence is an effective tumor suppressor mechanism because it prevents senescent cells from multiplying [5]. However, because senescent cells have the ability to release factors that stimulate the proliferation of cancer cells, their accumulation during aging can promote the growth of tumors [4]. Purinergic signal transduction mechanisms are a complex intercellular signaling network that regulates various biological processes in living cells. Thus, the study of ATP and purine nucleotides' role in cell fate, influencing proliferation, differentiation, or apoptosis, and their potential role in malignant transformation is a fascinating field [6].

Volonte et al*.*, firstly showed that exposure of WI-38 human diploid lung fibroblasts to sublethal concentrations of hydrogen peroxide (H_2_O_2_) induced oxidative stress in the cells, as indicated by increased senescence-related β-galactosidase (SA-β-gal) activity, senescence-related cell morphology and induction of markers of senescence (phospho-p53, p21, p16, and γ-H2A.X), as well as decreased cell proliferation. The authors then investigated the involvement of P2R activation. After oxidative stress stimulation, senescent WI-38 cells had a markedly higher level of extracellular ATP. Incubation with apyrase, an enzyme that converts ATP to AMP through hydrolysis, reduced the oxidative stress-mediated premature senescence development, as did treatment with the non-selective P2R antagonist, pyridoxal-phosphate-6-azophenyl-20,40-disulfonic acid tetrasodium salt (PPADS). In contrast, the adenosine P1 receptor antagonist, CGS 15943, had no effect. Additionally, exposure to ultraviolet C light accelerated the release of ATP and the induction of premature senescence. This suggests that P2R, but not P1 receptors are essential for oxidative stress-induced extracellular ATP-mediated premature senescence [2, 7].

This study then showed that in the absence of oxidative stress, ATP treatment was adequate to mediate premature senescence based on senescence-associated cell morphology accumulation, positive SA-β-gal activity and senescence marker up-regulation such as P-p53, p21, p16, and γ-H2A.X. Adenosine, however, did not induce WI-38 fibroblast senescence. Moreover, senescence was induced by ATPγS, a nonhydrolyzable analogue of ATP, and ARL 67156, which inhibits the hydrolysis of ATP. Furthermore, apyrase diminished ATP-induced senescence, as did PPADS, but not CGS 15943. The study supports another finding that a high-fat diet can cause adipose tissue senescence, mediated by elevated ATP content, through brief exposure [8].

mRNA expression profiling of P2R showed that P2Y_11_R and P2X_4_R were the predominant P2R subtypes expressed in senescent WI-38 fibroblasts. It's interesting to note that connexin-43 and pannexin-1, which mediate cellular release of ATP [9], were also expressed in WI-38 fibroblasts. Volonte et al., then demonstrated that the P2Y_11_R-selective antagonist, NF-157, abolished both ATP- and ARL67156-induced senescence; however, 5-BDBD, a selective P2X4R antagonist, had no effect, indicating that the P2Y_11_R may be the primary regulator of extracellular ATP-induced senescence. Additionally, NF-157 blocked the inhibition of BrdU incorporation and oxidative stress-induced premature senescence of WI-38 cells caused by ATP stimulation. Crucially, premature senescence was also induced by the selective P2Y_11_R agonist, NF-546. Thus, the P2Y_11_R is essential for ATP-induced human diploid fibroblasts premature senescence [2].

Regarding the intracellular molecular mechanisms underlying effects of P2Y_11_R stimulation, the authors demonstrated that ATP-induced senescence, as indicated by upregulation of p21 and increased activity of SA-β-gal, were abolished by U-73122, a phospholipase C (PLC) inhibitor, but were unaffected by SQ22536 or LRE-1, inhibitors of membrane and soluble adenylyl cyclase, respectively, indicating that PLC plays a crucial role in ATP/P2Y_11_R-induced senescence. When thapsigargin was used to preemptively deplete intracellular Ca^2+^ stores, the rise in cytosolic Ca^2+^ elicited by either ATP or the P2Y_11_R agonist, NF-546, was prevented. Moreover, ATP-induced premature senescence was inhibited by BAPTA-AM (an intracellular Ca^2+^ chelator). Thus, premature senescence of human fibroblasts was caused by intracellular Ca^+2^ release from the ER subsequent to ATP-initiated and P2Y_11_R/PLC-mediated signaling [2, 10].

It is well recognized that mitochondrial Ca^2+^ entry involves the mitochondrial Ca^2+^ uniporter (MCU). This study demonstrated that treatment of WI-38 fibroblasts with MCU inhibitors, Ru360 or KB R7943 mesylate, inhibited mitochondrial ROS production, in addition to inhibiting p21 overexpression and senescence-linked SA-β-gal activity. Also, ATP-induced upregulation of p21 was inhibited by siRNA-mediated MCU downregulation. These data collectively indicate that P2Y_11_R activation by extracellular ATP is a major cause of premature senescence, with ER-to-mitochondria signaling being a major contributing factor [2, 11].

Finally, the authors showed that ATP-mediated senescence stimulated growth of cancerous cells, as culturing the triple-negative breast cancer MDA-MB-231 cell line in conditioned media obtained from senescent WI-38 fibroblasts subsequent to H_2_O_2_ stimulation, induced cell proliferation and increased BrdU incorporation compared with when they were cultured in media obtained from control WI-38 cells. Moreover, this effect was abolished if the WI-38 cells were exposed to oxidative stress combined with apyrase treatment. Furthermore, this study showed that ATP-stimulated senescent WI-38 fibroblasts release a protumorigenic factor, amphiregulin, in response to P2Y_11_R stimulation, which was abolished by NF-157. Incubation of the conditioned media with an amphiregulin-neutralizing antibody also inhibited the MDA-MB-231 cell growth. These data indicate that through activation of P2R-mediated signaling within the tumor cells, ATP in the tumor microenvironment can promote the proliferation, survival, and potential for metastasis of cancer cells [12–15].

To summarize, this work provides evidence that extracellular ATP, acting at P2Y_11_Rs, can induce Ca^2+^ release from the ER and subsequent mitochondrial Ca^2+^ uptake to promote ROS generation, which induces premature senescence in human fibroblasts under oxidative stress. This study provides mechanistic evidence of a signaling cascade including ATP/P2Y_11_R/ER-Ca^+2^/ROS production and subsequent premature senescence and tumorigenesis.

## Data Availability

No datasets were generated or analysed during the current study.
